# The Effects of Neuropsychiatric Symptom Clusters in People with Dementia on Family Caregiver Burden

**DOI:** 10.3233/JAD-230972

**Published:** 2024-08-13

**Authors:** Robin van den Kieboom, Liselore Snaphaan, Ruth Mark, Marcel van Assen, Inge Bongers

**Affiliations:** a Tranzo, Tilburg School of Social and Behavioral Sciences, Tilburg University, Tilburg, The Netherlands; b Archipel Zorggroep, Eindhoven, The Netherlands; c Research Unit Evidence Based Management of Innovation, Mental Health Care Institute Eindhoven, Eindhoven, The Netherlands; d Department of Cognitive Neuropsychology, Tilburg School of Social and Behavioral Sciences, Tilburg University, Tilburg, The Netherlands; e Department of Methodology and Statistics, Tilburg School of Social and Behavioral Sciences, Tilburg University, Tilburg, The Netherlands; f Department of Sociology, Utrecht University, Utrecht, The Netherlands

**Keywords:** Alzheimer’s disease, behavioral and psychological symptoms of dementia, carers, community care, dementia

## Abstract

**Background:**

Neuropsychiatric symptoms are a robust risk factor for caregiver burden in family dementia caregivers. By grouping these symptoms, clinical interpretations regarding neuropsychiatric symptoms may facilitated because different groups of symptoms may require a different approach for intervention, thereby reducing caregiver burden.

**Objective:**

As clustering of neuropsychiatric symptoms could be clinically relevant, we aimed to explore the effects of these clusters on burden in family dementia caregivers.

**Methods:**

152 family dementia caregivers were included. Caregiver burden was measured using the Ervaren Druk door Informele Zorg (EDIZ)/Self-Perceived Pressure from Informal Care, a Dutch questionnaire. Caregivers also reported the neuropsychiatric symptoms and functional impairments in daily activities of the people with dementia they cared for. Multiple regression analyses were used in this cross-sectional study.

**Results:**

Adjusted for functional impairments and sociodemographic variables, neuropsychiatric symptoms were associated with more caregiver burden (*p* < 0.001). However, this association did not differ between the three neuropsychiatric symptom clusters (*p* = 0.745).

**Conclusions:**

Neuropsychiatric symptoms were associated with more family caregiver burden, but no conclusive evidence was found that this association differed for the three clusters. Clustering of neuropsychiatric symptoms is, however, worth exploring further in future studies with more participants. If specific links are found, these could be targeted in clinical practice in order to prevent, reduce and/or postpone caregiver burden.

## INTRODUCTION

Dementia is an umbrella term for several diseases that interfere significantly with a person’s ability to maintain the activities of daily living.^1^ The independence of people with dementia declines over time^2^ and more supervision and support are required,^3^ mostly by their family caregivers.^4^ Although family caregivers can and often do report positive aspects of caregiving such as feelings of mutuality or gratification,^5^ burden is probably more predominant.^6^ This negative impact of caring for a person with dementia is known as caregiver burden and is a complex, multidimensional construct.^7^

Caregiver burden is influenced by many factors including impairments in activities of daily living (ADL) and/or instrumental activities of daily living (IADL) and by the neuropsychiatric symptoms in people with dementia.^7^ These neuropsychiatric symptoms are frequently present^8^ and tend to increase as the dementia progresses.^9^ These symptoms tend to co-occur and are highly correlated with each other, such as among the symptoms of depression and anxiety or delusions and hallucinations. As a result, research has focused on clustering these symptoms, to help clarifying the underlying mechanisms, as different groups of symptoms may require a different approach for intervention in clinical practice.^10^ As such, the study of neuropsychiatric clusters has been classed as an area of importance by the Alzheimer’s Association.^11^ However, the neuropsychiatric manifestations of dementias are characterized by a marked inter-individual variability, which makes an overall composition of specific neuropsychiatric symptom clusters difficult. As a result, multiple models of clustering have appeared in the literature (see for an overview^12^).

As multiple models of clustering have received support in the literature, the selection of a model as framework for this study is based on a confirmatory analysis,^13^ including all the previously-reported cluster structures of neuropsychiatric symptoms ranging from two^14,15^ to three^16,17^— and four— cluster models, were compared.^17–19^ Two models fulfilled the criteria of excellent fit; the three- cluster model and four-cluster model of Sayegh and Knight.^17^ The other models did not meet the criteria for an excellent fit.^13^ In case of similar model fit the more parsimonious model is generally preferred.^13^ Therefore, the three-cluster model was selected as framework for this study, being the most parsimonious model of the two models with an excellent fit. The three-cluster model of Sayegh and Knight^17^ includes the clusters: *Hyperactivity* (agitation, disinhibition and irritability), *Affective* (depression, anxiety, sleep problems, appetite problems and apathy), and *Psychosis* (delusions and hallucinations).

Few studies, however, have so far explored the relationship between these neuropsychiatric clusters and caregiver burden and/or used different models of clustering.^20,21^ Cheng and colleagues^20^ found that a disruptive behavior cluster (i.e., agitation/aggression, irritability, disinhibition, aberrant motor behavior) was the strongest and most consistent predictor for caregiver burden followed by the mood cluster (e.g., anxiety, depression). Kim and colleagues’ found that that a psychosis symptom cluster (e.g. hallucinations, anxiety, euphoria, delusions and depression) was most influential with caregiver burden using a three-cluster model.^21^

To date, no study has explored the effects of the different neuropsychiatric clusters on caregiver burden using the three-cluster model of Sayegh and Knight’s model,^17^ which allegedly provides the most appropriate framework of clustering the neuropsychiatric symptoms.^13^ By using this framework, this study aims to explore whether adding neuropsychiatric symptom clusters results in a more suitable predictive model for caregiver burden and which cluster affects burden most, such that a specific treatment and support approach can be employed.

## METHODS

Data from the Innovate Dementia study was used (see protocol for further details^22^). This study was carried out in agreement with the Declaration of Helsinki. All participants signed an informed consent form.

This study protocol was reviewed and approved by the Medical Ethical Board of Maxima Medical Hospital (N19.027) approved the “Innovate Dementia protocol” in April 2019.

### Inclusion and exclusion criteria

People with a clinical diagnosis of dementia (any subtype), diagnosed by a healthcare specialists such as a neurologist or geriatrician. This diagnosis is based on the state-of-the-art guidelines of their professional association and the ICD-10 guideline.

These people were living at home. There was a committed family caregiver (spouse, family member, or friend) for the person with dementia and both the person with dementia and family caregiver had a sufficient understanding and mastery of the Dutch language.

### Procedure

Participants were recruited in the Eindhoven region from Alzheimer Nederland cafes, memory clinics in hospitals, elderly federations, and a mental health care institution (see protocol for further details^22^). When family caregivers responded to the flyers, they were contacted by the researchers and detailed information of the study was provided. When participants decided to collaborate, written informed consent was obtained from both the person with dementia and their family caregiver during a home visit. The questionnaires were sent digitally or by post to this convenience sample within a week after they had signed the informed consent forms. The questionnaires were filled in by the family caregiver.

### Variables and instruments

The following sociodemographic information was used regarding the people with dementia: age in years, gender, living situation (alone, with spouse, with spouse and children), educational level according to Verhage^23^ recoded as low (1– 4), medium (5), or high (6– 7). In addition, family caregivers’ age, gender, and their relationship (spouse, child or other (e.g., friend or acquaintance) with the person with dementia were obtained.

Caregiver burden was assessed using the Dutch questionnaire Ervaren Druk door Informele Zorg (EDIZ)/Self-Perceived Pressure from Informal Care.^24^ The EDIZ measures the caregivers appraisal of the perceived pressure regarding the demands of the caregiving situation. The EDIZ consists of nine statements, such as “I must always be available for my...”, with a family caregiver rating these statements on a 5-point Likert scale ranging from No! to Yes! According to the questionnaire manual, the scores are subsequently dichotomized into 0 (‘No!’ and ‘No’) and 1 (‘Yes!’, ‘Yes’ and ‘More or less’). This self-perceived pressure from informal care is seen as a one-dimensional latent variable, varying from less to more pressure. The total score ranges from 0 to 9, (0– 3 = low burden, 4– 6 = moderate burden, 7– 9 = high burden).^24^ In this study, the EDIZ had a good reliability with a Cronbach’s alpha of 0.81.

The frequency and severity of neuropsychiatric symptoms in the people with dementia were evaluated using the Neuropsychiatric Inventory Questionnaire (NPI-Q).^25^ The NPI-Q is highly correlated with the Neuropsychiatric Inventory and provides a brief, reliable, informant-based assessment.^25^ The NPI-Q was chosen to reduce time and burden on the participants. NPI-Q consists of 12 items, representing the different neuropsychiatric symptoms. Each item is scored on presence (Yes = 1 or No = 0). Subsequently, for each item marked as present, severity is scored on a three-point scale (Mild = 1, Moderate = 2, Severe = 3). The total score of the NPI-Q has a range of 0 to 36. The Cronbach’s alpha was 0.75 in the current study. Sayegh and Knight’s model^17^ was used to group the symptoms into three clusters, namely: *Hyperactivity* (agitation, disinhibition and irritability) (range 0– 9), *Psychosis* (delusions and hallucinations) (range 0– 6), and *Affective* (depression, anxiety, sleep problems, appetite problems and apathy) (0– 15). Cronbach’s alpha for the different clusters were 0.66, 0.43, 0.60, respectively.

Functional impairments (ADL) were evaluated by the Katz Index of Independence in Activities of Daily Living (KATZ).^26^ Each of the six items were scored on four-point scale (1 = totally independent, 2 = limited help needed, 3 = extensive help needed, 4 = totally dependent). The total score ranges from 6 to 24, with higher scores representing more functional dependency. In this current study, the KATZ had a Cronbach’s alpha of 0.85.

The Lawton-Brody Instrumental Activities of Daily Live scale^27^ was used to assess the ability of the person with dementia on instrumental activities of daily living (IADL). The total score ranges from 0 (low function, dependent) to 8 (high function, independent). In this current study, the Cronbach’s alpha of the IADL questionnaire was 0.70.

### Statistical analysis

Descriptive statistics were used to summarize the demographics and clinical characteristics of the people with dementia and caregivers. The NPI-Q was computed as a ten-items total score, by excluding the items of euphoria and repetitive behavior as these items are not included in the three clusters. A multiple linear regression analysis was used to assess the effects of adding the clusters of neuropsychiatric symptoms (NPI-Q) on caregiver burden, adjusted for the total score of the NPI-Q, sociodemographic variables and functional impairments in activities of daily living (ADL) and instrumental activities (IADL). In the first block, sociodemographic variables of both caregiver and the person with dementia were entered in the regression analysis. In the second block IADL and ADL were added. In the third block the total score of the NPI-Q was included. In the final block, total NPI-Q was replaced by the three NPI-Q clusters (*Psychosis* & *Hyperactivity* & *Affective)* to assess whether the three separate clusters provided a better prediction of caregiver burden than merely the total score of the NPI-Q.

We focused on testing and interpreting the incremental explained variance of caregiver burden (*ΔR*^2^) of each block, and the effects of predictors in the best fitting model (third block with total score of the NPI-Q or fourth block with three clusters).

## RESULTS

### Sample description

The data from 152 dyads, all questionnaires filled in by the family caregiver, were used. The demographics of the people with dementia and their family caregivers are presented in Table 1. Most of the people with dementia were female (*n* = 85, 56.7%), and living with their spouse (*n* = 74, 54.4%), and had a low education (*n* = 72, 50.7%). Age of the people with dementia ranged between 53 and 97 years (mean 78.77, sd = 8.62). Furthermore, 94.6% (*n* = 141) had at least one neuropsychiatric symptom, while on average, five neuropsychiatric symptoms were simultaneously present. 56.3% of the people with dementia, experienced at least one symptom of the *Affective* cluster, 44.4% of the *Psychosis* cluster and 45.0% of the *Hyperactivity* cluster (Table 2). Furthermore, 6.0% of them had no symptoms included in any of the three clusters (aberrant motor behavior and euphoria were not included in the three-cluster model), 20.0% only had symptom(s) in one cluster, and 74.0% had symptoms in two or three clusters. Most of the family caregivers were female (*n* = 100, 67.6%), were spouses (*n* = 77, 51.7%) and experienced moderate levels of burden (mean = 5.41, sd = 2.55). Age of the informal caregivers ranged between 24 and 93 years (mean 73.87, sd = 13.47).

**Table 1:** Demographics and clinical characteristics of the people with dementia and caregivers

Variable (n)	Mean (SD)	Frequency (%)
People with dementia (*n* = 152)
Gender (*n* = 150)
Female (*n* = 85)		56.70%
Male (*n* = 65)		43.30%
Age in years (*n* = 144) range (53– 97)	78.77 (8.62)	
Education^1^ (*n* = 142)
Low (*n* = 72)		50.70%
Medium (*n* = 33)		23.20%
High (*n* = 37)		26.10%
Living situation (*n* = 136)
Alone (*n* = 52)		38.20%
With spouse (*n* = 74)		54.40%
With spouses and children (*n* = 10)		7.40%
NPI Presence of symptoms (*n* = 149)		94.60%
NPI Number of symptoms presence (*n* = 150)	4.99 (2.66)
NPI Total score (range 0– 36) (*n* = 150)	9.39 (6.35)
KATZ Total score (range 6– 24) (*n* = 148)	8.55 (3.33)
Lawton Total score (range 0– 8) (*n* = 151)	3.34 (1.48)
Caregiver (*n* = 152)
Gender (*n* = 148)
Female (*n* = 100)		67.60%
Male (*n* = 48)		32.40%
Age in years (*n* = 144) range (24– 93)	63.87 (13.47)
Relation (*n* = 149)
Spouse (*n* = 77)		51.70%
Child (*n* = 61)		40.90%
Other (*n* = 11)		7.40%
EDIZ Total score (range 0– 9) (*n* = 152)	5.41 (2.55)

^1^Educational level according to Verhage^23^, recoded as low (1–4), medium (5), or high (6–7).

**Table 2:** Frequency of the neuropsychiatric symptoms in our sample clustered according to the three-cluster model of Sayegh and Knight^17^

NPI Clusters *n* = 151	Frequency (%)
Affective (*n* = 85)	56.3%
Psychosis (*n* = 67)	44.4%
Hyperactivity (*n* = 68)	45.0%
Presence of number of NPI clusters (*n* = 150)
0 (*n* = 9)	6.0%
1 (*n* = 30)	20,0%
2 (*n* = 58)	38.7%
3 (*n* = 53)	35.3%

### The effect of neuropsychiatric symptoms on caregiver burden

In Table 3, the correlations between the three neuropsychiatric symptoms clusters are presented. As shown, the clusters have small to medium correlations. In Table 4 the correlations between the individual neuropsychiatric symptoms are presented. Most symptoms have small to medium correlations to each other, as well within and outside the clusters.

**Table 3:** Correlations of the neuropsychiatric symptoms clusters on the NPI-Q

	Hyperactivity cluster	Affective cluster	Psychosis cluster
Hyperactivity cluster	1	–	–
Affective cluster	*r* (149) = 0.482	1	–
	*p* < 0.001*
Psychosis cluster	*r* (149) = 0.420	*R* (149) = 0.318	1
	*p* < 0.001*	*p* < 0.001*

^*^Significant at the 0.01 level (2-tailed).

**Table 4:** Correlations of the individual neuropsychiatric symptoms on the NPI-Q

	Delusions^p^	Hallucinations^p^	Agitation^h^	Depression^a^	Anxiety^a^	Apathy^a^	Disinhibition^h^	Irritability^h^	Sleep problems^a^	Appetite problems^a^
Delusions^p^	1	–	–	–	–	–	–	–	–	–
Hallucinations^p^	*r*(146) = 0.309	1	–	–	–	–	–	–	–	–
	*p* < 0.001**
Agitation^h^	*r*(143) = 0.405	*r*(144) = 0.128	1	–	–	–	–	–	–	–
	*p* < 0.001**	*p* = 0.126
Depression^a^	*r*(146) = 0.139	*r*(147) = 0.097	*r*(145) = 0.282	1	–	–	–	–	–	–
	*p* = 0.094	*p* = 0.241	*p* < 0.001**
Anxiety^a^	*r*(144) = 0.322	*r*(145) = 0.203	*r*(143) = 0.211	*r*(146) = 0.272	1	–	–	–	–	–
	*p* < 0.001**	***p*** = 0.014*	***p*** = 0.011*	*p* < 0.001**
Apathy^a^	*r*(146) = 0.183	*r*(147) = 0.126	*r*(144) = 0.283	*r*(147) = 0.380	*r*(145) = 0.381	1	–	–	–	–
	***p*** = 0.027*	*p* = 0.128	*p* < 0.001**	*p* < 0.001**	*p* < 0.001**
Disinhibition^h^	*r*(146) = 0.335	*r*(147) = 0.121	*r*(144) = 0.418	*r*(147) = 0.142	*r*(145) = 0.173	*r*(147) = 0.268	1	–	–	–
	*p* < 0.001**	*p* = 0.146	*p* < 0.001**	*p* = 0.087	***p*** = 0.038*	***p*** = 0.001**
Irritability^h^	*r*(144) = 0.325	*r*(146) = 0.157	*r*(142) = 0.381	*r*(145) = 0.188	*r*(143) = 0.406	*r*(145) = 0.333	*r*(145) = 0.430	1	–	–
	*p* < 0.001**	*p* = 0.058	*p* < 0.001**	***p*** = 0.024*	*p* < 0.001**	*p* < 0.001**	*p* < 0.001**
Sleep problems^a^	*r*(141) = 0.118	*r*(141) = 0.187	*r*(137) = 0.131	*r*(140) = 0.181	*r*(138) = 0.251	*r*(140) = 0.273	*r*(140) = 0.1510	*r*(139) = 0.365	1	–
	*p* = 0.163	***p*** = 0.027*	*p* = 0.128	***p*** = 0.033*	***p*** = 0.003*	***p*** = **0.001**	*p* = 0.075	*p* < 0.001**
Appetite problems^a^	*r*(141) = 0.248	*r*(141) = 0.064	*r*(139) = 0.180	*r*(142) = 0.145	*r*(140) = 0.165	*r*(141) = 0.287	*r*(142) = 0.250	*r*(139) = 0.186	*r*(137) = 0.245	1
	***p*** = 0.003*	*p* = 0.454	***p*** = 0.034*	*p* = 0.086	*p* = 0.052	*p* < 0.001**	***p*** = 0.003*	***p*** = 0.028*	***p*** = 0.004*

^p^Psychosis cluster; ^h^Hyperactivity cluster; ^a^Affective cluster. ^*^ Significant at the 0.05 level (2-tailed). ^**^Significant at the 0.01 level (2-tailed).

The results of the multiple regression analyses are shown in Table 5. Demographic variables affected caregiver burden (*R*^2^ = 0.133, *F*(10,126) = 1.934, *p* = 0.046, Cohen’s *f*^2^ = 0.15). Adjusted for the sociodemographic variables, IADL and ADL improved the prediction of burden (*ΔR*^2^ = 0.063, *F*(2,124) = 4.860, *p* = 0.009, Cohen *f*^2^ = 0.07). Subsequently, adding the total NPI score of the ten items also improved the prediction of caregiver burden (*ΔR*^2^ = 0.083, *F*(1,123) = 14.109, *p* < 0.001, Cohen *f*^2^ = 0.09). The model with the total NPI score explained 27.9% of the variance of caregiver burden. Finally, controlling for aforementioned predictors, replacing the total NPI score with the three clusters of neuropsychiatric symptoms (Psychosis (*B* = 0.243, *p* = 0.105) and Affective (*B* = 0.138, *p* = 0.063 and Hyperactivity (*B* = 0.089, *p* = 0.376) showed no improvement in the predictive model of caregiver burden (*ΔR*^2^ = Cohen’s *f^2^* = 0.004, *F*(2,121) = 0.295, *p* = 0.745), concluding that the effect on caregiver burden was similar across the three clusters.

**Table 5:** Multiple regression analysis of caregiver burden with sociodemographic variables, IADL and ADL, total NPI score and the three neuropsychiatric clusters of the NPI

	B [95% CI]	*se*	*p*
Block 1: socio-demographics
Age caregiver	0.014 [– 0.050, 0.077]	0.032	0.666
Age person with dementia	0.019 [– 0.050, 0.088]	0.035	0.583
Gender caregiver (male)	– 0.213 [– 1.153, 0.727]	0.475	0.655
Gender person with dementia (male)	0.295 [– 0.691, 1.281]	0.498	0.555
Relation (child)	– 0.204 [– 2.305, 1.897]	1.061	0.848
Relation (other)	– 2.855 [– 5.309, – 0.402]	1.239	**0.023**
Education person with dementia (moderate)	0.263 [– 0.708, 1.235]	0.491	0.593
Education person with dementia (high)	– 0.746 [– 1.726, 0.234]	0.495	0.135
Living situation person with dementia (with spouse)	0.949 [– 0.799, 2.698]	0.883	0.285
Living situation person with dementia (with spouse and child)	– 0.224 [– 1.250, 0.802]	0.518	0.666
*R*^2^ = 0.133	*F*(10,126) = 1.934, *p* = **0.046**
Block 2: functional impairments
ADL	0.165 [0.008, 0.322]	0.080	**0.040**
IADL	0.232 [– 0.085, 0.548]	0.160	0.150
*ΔR*^2^ = 0.063	*F*(2,124) = 4.860, *p* = **0.009**
Block 3: Total sum of neuropsychiatric symptoms
Total score NPI (10 items)	0.139 [0.066, 0.212]	0.037	**<0.001**
*ΔR*^2^ = 0.083	*F*(1,123) = 14.109, *p* < **0.001**
*R*^2^ total = 0.279	*F*(13,123) = 3.658, *p* < **0.001**

*B*, *se*, and *p* are unstandardized regression coefficient, its standard error, and the two-tailed *p*-value of the *t*-test of *B*. *p*-values below 005 are printed in bold. *R*^2^ is the proportion of explained variance of the regression model, *ΔR*^2^ is the change in *R*^2^ after adding the predictors of one block to the model.

Focusing on the effects of individual predictors (as shown in Table 5), of all sociodemographic variables, only type of relation was associated to burden. Spouses experienced more burden compared to other caregivers (*B* = – 2.855, *p* = 0.023), but not more than adult child caregivers (*B* = – 0.204, *p* = 0.848). Functional impairments in ADL activities, such as bathing and dressing, were associated with more caregiver burden (*B* = 0.165, *p* = 0.040). Dependency in IADL activities, such as shopping or housekeeping, was not associated to experiencing burden (*B* = 0.232, *p* = 0.150). The total score of the ten items on the NPI was associated with more burden (*B* = 0.139, *p* < 0.001).

## DISCUSSION

Neuropsychiatric symptoms are consistent and robust risk factors for caregiver burden^7^ and previous studies explored different models to cluster these symptoms.^13^ However, limited and inconsistent results are reported regarding the effects of these neuropsychiatric symptom clusters on family caregiver burden. Therefore, this study aimed to explore whether adding different clusters, by using the three-cluster model of Sayegh and Knight^17^ as framework, provided a better predictive model for caregiver burden than a model with the total sum of neuropsychiatric symptoms. In our study, neuropsychiatric symptoms were very prevalent (found in almost 95% of the people with dementia) with an average of five simultaneous symptoms per person. This is in line with percentage rates reported in previous studies.^28^

Our results showed that more neuropsychiatric symptoms were associated with more burden, but we found no evidence for a difference between the three clusters. These findings are in contrast with previous studies^20,21^ although an accurate comparison is complex due the different clustering models. The study of Cheng^20^ is most comparable and used a four-cluster model with a *Behavioral* (agitation, disinhibition, irritability, aberrant motor behavior), *Psychosis* (delusions and hallucinations), *Mood* (depression, anxiety, sleep, appetite, apathy) and an *Euphoria* cluster (euphoria). They found in a sample of people with mild to moderate Alzheimer’s disease that the *Behavioral* and *Mood* clusters had an effect on caregiver burden. A possible explanation for this difference, is that more family caregivers reported behavioral (72%) and mood symptoms (63%) in the study of Cheng et al.^20^ compared to this current study. This current study also used the three-cluster model of Sayegh and Knight,^17^ in which the *Hyperactivity* cq. *Behavioral* cluster does not include aberrant motor behavior.

In the study of Kim and colleagues,^21^ three symptom clusters were used in a sample of people diagnosed with or suspected for Alzheimer’s dementia. The *Hyperactivity* cluster (disinhibition, irritability, and agitation), *Psychosis* cluster (hallucinations, anxiety, elation/euphoria, delusions, and depression) and the *Physical Behavior* cluster (appetite and eating abnormalities, apathy/indifference, aberrant motor behavior, sleep, and night-time behavior disturbances). They found that each of these clusters were associated with caregiver burden, with the *Psychosis* cluster most influential. This study assessed caregiver burden by rating the emotional distress for each of the individual neuropsychiatric symptoms. However, the emotional distress scale was developed as a measure of caregiver distress in relation to neuropsychiatric symptoms, and not to investigate caregiver burden or distress per se.^29^ Besides, caregiver burden is influenced by many patient-, caregiver- and context-factors^7^ and not solely by neuropsychiatric symptoms. Both the studies of Cheng^20^ and Kim^21^ used the NPI, which differs slightly from the NPI-Q used in this current study. Although both questionnaires have the same domains of neuropsychiatric symptoms, the NPI also has a frequency scale for each symptom besides the severity scale, where the NPI-Q only measures the presence of a symptom. Therefore, the NPI has a total range from 0 to 144, whereas the NPI-Q has a range from 0 to 36, which makes comparison between the previous studies and the current study not completely analogous. However, as both scales use the exactly the same 12 domains, we expect similar results with boththese scales.

We found no conclusive evidence for the differentiation between the effect of different clusters on caregiver burden. There are several possible explanations for these null findings;

(i) The null findings are true and distinguishing clusters does not improve the prediction model of caregiver burden. That is, the presence of specific clusters of symptoms do not make informal caregivers less or more at risk for caregiver burden. In that case, clinical practice should, focus on the total NPI-Q score.
(ii) Clustering of symptoms results in a more predictive model of burden but with a small effect size, however this current study had null findings due to a lack of statistical power to detect this small effect. Using G*Power^30^ we calculated that our test (block 4, Table 3, based on *N* = 137) had a power of 0.985 to detect a medium effect size (*f^2^* = 0.15), but a mere power of 0.29 to detect a small effect size (*f^2^* = 0.02). Hence if the effect on caregiver burden slightly differs across clusters, the present data were probably not powerful enough to detect it. Future research should use the same three-cluster model^17^ with a larger sample of participants to explore the differentiation of the effects of the clusters on burden.
(iii) Clustering of symptoms could result in more predictive model of burden, but by including many covariates we limited the statistical power of our analyses. Although we indeed incorporated multiple covariates, re-analyzing the data with only including age and gender of the informal caregiver as covariates did also not result in an improvement of the prediction of burden with three clusters of symptoms.
(iv) Clustering of symptoms could result in a more predictive model of burden, but the selected model of three clusters^17^ may not be the most suitable model. Future studies with larger sample sizes could potentially compare different models with the same dataset and in this way explore which model fits best.

It should be noted that the study of Liew^13^ included a large sample of people (*n* = 8,530) with mild cognitive impairments (MCI) to explore the clusters of neuropsychiatric symptoms.^13^ The manifestation of neuropsychiatric symptoms differs throughout the spectrum of MCI and mild to severe dementia,^31^ therefore it is debatable whether the three-cluster model of Sayegh and Knight^17^ is the most appropriate framework for clustering neuropsychiatric symptoms in people with dementia. For example, the four-cluster model from Aalten and colleagues^18^ was based on a large sample of people with Alzheimer’s dementia and found the clusters *Hyperactivity* (agitation, disinhibition, irritability, motor disturbance), *Affective* (depression, anxiety), *Apathy* (apathy, appetite) and *Psychosis* (delusions, hallucinations, sleep).

Although the predictive abilities of neuropsychiatric symptoms clusters may help to guide the practices of dementia care management, there is uncertainty regarding the use of neuropsychiatric symptom clusters in relation to caregiver burden. A review of Canevelli and colleagues showed a relatively low concordance among neuropsychiatric clusters, as no pair of studies reported the same cluster composition.^32^ Besides, rather than symptoms that correlate strongly within the cluster and weakly with symptoms outside the cluster, most symptoms have small to medium correlations to each other as shown in Table 4. Consequently, (i) symptoms do not systematically group into the same clusters in different studies, and (ii) the absence of a cluster of strongly correlated symptoms causes only a small true improvement of fit relative the NPI total score, which is hard to detect when not having a (very) large sample sizes (i.e., low statistical power). Moreover, a three-years longitudinal study^33^ showed that neuropsychiatric symptoms cannot be neatly partitioned into clusters that are stable over time. This in line with findings that neuropsychiatric symptoms fluctuate over time.^34^ Previous studies did find different effects of clusters,^20,21^ so future research should focus on better characterizing the relationships between pairs or groups of symptoms on caregiver burden and identifying shared causal underpinnings, as mentioned by Connors and colleagues.^33^

A limitation of this current study that people with a possible prior history of psychiatric symptoms were not excluded in the analysis, as this history was not assessed in the original Innovate Dementia study.^22^ Therefore, it is possible that for some people with dementia and their informal caregivers, the psychiatric symptoms and the resulting caregiver burden were unrelated to dementia but already a pre-existing predisposition. Therefore, we recommend future studies to assess the history of psychiatric symptoms in the person with dementia at baseline measurement.

In this study we focused on the effect of the different neuropsychiatric symptom following the three-cluster model of Sayegh and Knight^17^ on caregiver burden, adjusted for the functional dependence of the person with dementia. However, caregiver burden is impacted by many other factors,^7^ for example being the sole caregiver, time since diagnose, sense of competence of the informal caregiver, but also the presence of professional support services.^35,36^ Unfortunately, we were not able to include these factors in our predictive model of caregiver burden. Future research is needed with sufficient statistical power, in order to explore the potential of neuropsychiatric symptom clusters on burden, controlled for the different factors of the person with dementia, informal caregiver and the caregiving situation.

To conclude, neuropsychiatric symptoms were associated with more family caregiver burden, but this study found no conclusive evidence that this association differed for the three clusters. Clustering of neuropsychiatric symptoms is, however, worth exploring further in future studies with more participants and different models of clustering. If specific links are found, these could be targeted in clinical practice in order to prevent, reduce and/or postpone caregiver burden.

## AUTHOR CONTRIBUTIONS

Robin van den Kieboom (Conceptualization; Formal analysis; Methodology; Writing – original draft); Liselore Snaphaan (Conceptualization; Methodology; Supervision; Writing – review & editing); Ruth Mark (Conceptualization; Supervision; Writing – review & editing); Marcel van Assen (Formal analysis; Writing – review & editing); Inge Bongers (Conceptualization; Supervision; Writing – review & editing).

## Data Availability

The data will not be made public, assuring the study participants’ privacy. Request for data sharing will be considered on an individual basis, for appropriate research purposes only.
